# Ophthalmology

**Published:** 1897-05

**Authors:** Alvin A. Hubbell


### Progress in Medical Science.

### OPHTHALMOLOGY.

Conducted by ALVIN A. HUBBELL, M. D.

### HOLOCAIN—A NEW LOCAL ANESTHETIC.

GUTMANN (*Deut. Med. Woch.*, March 11, 1897,) relates his clinical investigations with this new anesthetic, which is closely allied to phenacetin. The chloride of holocain crystallises in white needles, dissolves in hot water, but is only sparingly soluble in cold water. If the salt is heated with water in a glass vessel the solution is often cloudy, owing to a small amount of alkali present; hence a porcelain vessel should be used. The author first records his experiments upon rabbits. He has used it in thirty cases in men, of which thirteen were examples of a foreign body in the eye, two of keratitis, seven of operations on the eye and in eight cases the eyes were healthy. One minute after the introduction of three to five drops of a 1 per cent. solution there was anesthesia of the cornea. A passing, burning sensation was felt. The foreign bodies were removed without inconvenience, and the galvano-cautery was used in a case of corneal ulcer and in a case of keratitis. Tattooing was also performed in two cases of leucoma. In one case it was possible to compare tenotomy of the eye muscles under cocaine and under holocain. Less pain was experienced in the latter instance. The duration of anesthesia under holocain is from five to fifteen minutes. The cornea remains moist and shining. The ocular tension is not diminished. The pupil remains unaltered and accommodation is unaffected. In a patient aged forty-eight three drops of a 2 per cent. solution of cocaine were introduced into one eye; anesthesia appeared in two and a half minutes, and lasted three and a half minutes, the pupil dilated and the tension diminished. With three drops of a 1 per cent. solution of holocain introduced into the other eye anesthesia appeared after a slight feeling of burning one minute and lasted nine minutes; the pupil was not dilated and there was no alteration in tension. The rapidity of anesthesia with holocain is an advantage. The dilatation of the pupil under cocaine for the removal of foreign bodies is a disadvantage. The lessened pressure is a drawback in cataract extraction, but useful in operations for glaucoma. The author says that holocain should not be used subcutaneously, as intoxication symptoms were rapidly produced by a small dose in a rabbit. For external use he recommends it as a substitute for cocaine.—*British Medical Journal*, April 3, 1897.

---

EUCAINE.

L. S. Somers, (*Therap. Gaz.*, January 15th,) has recently used eucaine in a series of cases of hypertrophic nasal catarrh in place of cocaine to produce anesthesia of the enlarged turbinals, so that reduction in their size by cauterisation might be effected. A 4 per cent. solution of eucaine hydrochlorate in distilled water was used, this corresponding to the strength of cocaine solution previously employed for that purpose. The drug was applied to the turbinated bodies in the same manner as cocaine, by saturating a cotton plug with a definite quantity of the solution and applying it to the part to be operated on until anesthesia was complete. The cauterisation was effected by means of chromic acid crystals melted on the end of an appropriate applicator by means of the alcohol lamp. The results obtained can better be expressed by comparing the effects of cocaine and eucaine *seriatim.* Cocaine usually produces its anesthetic effects in the nasal cavity in from three to five minutes, anesthesia continuing for twenty minutes to half an hour. In the series of cases in which eucaine was used, anesthesia was not complete until eight or ten minutes had expired, and the loss of sensibility remained about twenty minutes. When an excessive amount of cocaine solution is used in the nares it occasionally flows backward into the nasopharynx, producing a very uncomfortable sensation of numbness in that region. It was noticed that this did not occur so markedly when eucaine was used, probably because of its slower anesthetic action. Eucaine causes hyperemia of the mucous membrane when locally applied. This action is directly opposite to that of cocaine and renders the drug of little value when the turbinal tissues are much hypertrophied, as the cauterising agent cannot be introduced into the nasal cavity without injuring the septum. This action of eucaine, in producing a temporary local congestion, militates greatly against the use of the drug in active inflammatory conditions, but may possibly be obviated by using equal parts of the eucaine and cocaine, as recommended by Berger. Eucaine is a poison similar to cocaine, but has the great advantage of being decidedly less harmful to the organism when used in the same doses. Another advantage which the drug possesses over cocaine is that it may be kept for an indefinite time in solution with sterilised water, 1 part to 10. The solution may be repeatedly sterilised by boiling without impairing its anesthetic properties.

Pouchet, at a meeting of the Société de Thérapeutique, (*Sem. Med.*, February 3d,) said that he had investigated the physiological action of eucaine, and had found that the toxic equivalent of that drug was almost equal to that of cocaine. Moreover, eucaine may produce toxic effects which may even prove fatal without any prodromic stage. It acts on the heart with an intensity equal, if not superior to, that of cocaine. In a frog in which 2 mg. of eucaine were injected, there was observed a considerable slowing of the heart's beat with arhythmia, whereas equal doses of cocaine produced no effect. Eucaine must, therefore, be looked upon as rather a dangerous anesthetic.

Reclus, who has studied the effects of eucaine from a clinical point of view, has satisfied himself that in equal doses its anesthetic power is less than that of cocaine, and he thinks therefore that it should not be used in serious operations.—*British Medical Journal*, February 27, 1897.

---

### THE APPLICATION OF THE RÖNTGEN RAYS IN THE DIAGNOSIS OF FOREIGN BODIES IN THE VITREUS.

DR. G. O. RING (*Transactions of College of Physicians of Philadelphia*) exhibited two radiographs, showing the presence and the approximate location in the vitreus of a piece of metal, and the patient upon whom Mule's operation had been performed.

Eighteen months previously a piece of steel had entered the right eye through the cornea, producing a tear in the iris, cataract, and complete blindness. The eye had become subject to attacks of ciliary inflammation, and recently the left eye had shown signs of sympathetic irritation. The steel, surrounded by exudate, could not be dislodged from its position in the ciliary body by the Hirschberg magnet, and evisceration was performed and the glass inserted. Very little reaction followed. In the discussion Dr. H. T. Hansell said that the case he reported in the discussion in December was exposed for eight minutes, and the skiagraph showed exactly the position of the foreign body in relation to the shadow of the bone ; but this picture demonstrated what his did not—namely, that the Röntgen rays will cast the shadow of a piece of metal lying in any part of the vitreus. This method is extremely valuable and now almost essential in the diagnosis of the presence and composition of foreign bodies. Dr. George Friebis said that he preferred decidedly evisceration to enucleation when feasible, and was gratified to find that the reaction may be insignificant. Dr. C. A. Oliver said that it must be remembered that should the vitreus contain blood-clots the magnet must be brought almost in contact with the metal before the latter can be moved from its position ; and, again, should the metal, during its passage through the vitreus, fall from the magnet, it may pursue a direction entirely different from that in which it had been carried by the magnet.

---

### A PIECE OF STEEL IMBEDDED IN THE CILIARY BODY LOCATED BY MEANS OF RÖNTGEN'S RAYS.

DR. DE SCHWEINITZ (*Transactions of College of Physicians of Philadelphia,* February 16, 1897), described a case in which extraction of a piece of steel was performed with the electro-magnet after two similar operative procedures without the use of the rays had been unsuccessful. In spite of the repeated traumatisms the patient recovered with good vision. The foreign body had remained in the ciliary body for twelve days and had caused cyclitis. Dr. Max J. Stern, of the Philadelphia Polyclinic, furnished the radiograph, which clearly indicated the position of the foreign body, and which had been unrecognisable by ophthalmoscopic examination, owing to the hazy condition of the media. In the discussion Dr. Hansell said that the case just reported was the fourth in Philadelphia, and with the exception of those of Williams, of Boston, and Clark, of Columbus, the only ones that have thus far appeared in print. Dr. de Schweinitz was to be congratulated on the brilliant success following the extraction of the steel. The condition of the eye indicated that still further improvement of vision was to be expected. It has been thus positively demonstrated that the Röntgen rays will not only penetrate the coats of the eye and the media, but also the bones of the orbit, and that both the presence and the approximate location of a piece of metal can be positively diagnosticated. Dr. Norris said that in confirmation of the last remark he had recently treated a case where Dr. Leonard has, by passing the rays through the bones of the head, demonstrated the presence of two No. 6 birdshot in the orbit and one in the pharynx in the same patient. The Crooke's tube was held on one side of the head and the plate fastened on the opposite temple. Dr. Wm. Thomson suggested the advisability of inserting into the conjunctival sac a strand of lead wire and the encasing of the anterior section of the ball in a lead plate as material aids in locating the precise position of the foreign body.

